# The Effectiveness of a Group Kickboxing Training Program on Sarcopenia and Osteoporosis Parameters in Community-Dwelling Adults Aged 50–85 Years

**DOI:** 10.3389/fmed.2022.815342

**Published:** 2022-04-25

**Authors:** Yen-An Lin, Lee-Hwa Chen, Fang-Ping Chen, Alice May-Kuen Wong, Chih-Chan Hsu, Jau-Yuan Chen

**Affiliations:** ^1^Department of Family Medicine, Chang Gung Memorial Hospital, Taoyuan City, Taiwan; ^2^Department of Athletic Training and Health, National Taiwan Sport University, Taoyuan City, Taiwan; ^3^Department of Obstetrics and Gynecology, Osteoporosis Prevention and Treatment Center, Keelung Chang Gung Memorial Hospital, Taoyuan City, Taiwan; ^4^College of Medicine, Chang Gung University, Taoyuan City, Taiwan; ^5^Department of Physical Medicine and Rehabilitation, Chang Gung Memorial Hospital at Taoyuan, Taoyuan City, Taiwan; ^6^Healthy Aging Research Center, Chang Gung University, Taoyuan City, Taiwan

**Keywords:** DXA, geriatric syndrome, exercise intervention, active aging, dynapenia

## Abstract

**Background:**

Sarcopenia and osteoporosis are important health issues faced by older people. These are often associated with each other and share common risk factors and pathologic mechanisms. In the recently revised consensus of the European Working Group on Sarcopenia in Older People, low muscle strength has been defined as the first characteristic of sarcopenia rather than a loss in muscle mass, and walking speed has been stated as an indicator of the severity of sarcopenia. It is believed that these markers of muscle function can be potentially reversed *via* exercise-based interventions. The purpose of this study was to evaluate the effects of kickboxing exercise training on the parameters of sarcopenia and osteoporosis in community-dwelling adults.

**Methods:**

In total, one hundred eligible subjects were randomized into an intervention group (*n* = 50) with 76% women and control group (*n* = 50) with 86% women. Both the intervention and control groups were provided with classroom lectures and personal consultations pertaining to sarcopenia and osteoporosis, whereas a 12-week kickboxing exercise training was arranged only for the intervention group. All anthropometric, physical performance, body composition, and bone mineral density measurements along with participant completed questionnaires were conducted before and after the training period.

**Results:**

After 12 weeks, 41 participants in the intervention group and 34 participants in the control group completed the final assessments. There was no difference between the intervention and control groups in terms of basic demographic data. The BMI (+1.14%) of the control group increased significantly during the study period. The waist circumference (−6.54%), waist-to-height ratio (−6.57%), waist–to–hip ratio (−4.36%), total body fat (−1.09%), and visceral fat area (−4.6%) decreased significantly in the intervention group. Handgrip strength (+5.46%) and gait speed (+5.71%) improved significantly in the intervention group. The lean body mass increased by 0.35% in the intervention group and by 0.9% in the control group. The femoral neck bone mineral density (−1.45%) and T score (−3.72%) of the control group decreased significantly. The intervention group had more improvement in the status of sarcopenia (OR 1.91) and osteoporosis over the control group. Finally, the intervention group had less deterioration in the status of sarcopenia (OR 0.2) and osteoporosis (OR 0.86) compared with the control group.

**Conclusion:**

Our study demonstrated that a 12-week kickboxing exercise training program is effective for improving sarcopenic parameters of muscle strength and function, but not muscle mass in adults, aged 50–85 years. Furthermore, markers of osteoporosis also showed improvement. These findings suggest that a 12-week kickboxing program is effective for muscle and bone health among community-dwelling older individuals.

## Introduction

Osteoporosis and sarcopenia are common musculoskeletal disorders in older people, and they are associated with significant morbidity and mortality (1). These conditions are linked to a high risk of falls, loss of autonomy, and an increased likelihood of being admitted to a nursing home for minor health problems. Bone mass, muscle mass, and muscular strength increase in early life but start to decrease noticeably in the fifth decade of life. Sarcopenia is defined as a low muscle mass combined with slow gait speed or weak hand grip power. Muscle strength plays more important than muscle mass, because dynapenia usually happens ahead of sarcopenia. Sarcopenia and osteoporosis are typical features of aging in the musculoskeletal system and are often associated with each other, sharing common risk factors and also the pathogenic mechanisms (2, 3); both are major contributors to disability and frailty associated with aging (4, 5). In addition to old age, inactivity and poor nutrition are important causes of sarcopenia as per the revised guidelines of the European Working Group on Sarcopenia in Older People (6). Among the three diagnostic criteria of sarcopenia, the updated consensus emphasizes low muscle strength as the key characteristic of sarcopenia rather than low muscle mass. Furthermore, physical performance, measured by usual walking speed, is used to identify the severity of sarcopenia because of its better prediction ability for adverse outcomes. The functional decline among many older adults starts as early as age 50 (7), and non-pharmacological methods can be used as potential treatment options in such cases. The evidence regarding exercise training for sarcopenia has been established more firmly than the evidence regarding the nutritional intervention (8). Strength- and weight-bearing aerobic exercise training has been proven to maintain or increase bone mineral density (9).

Kickboxing is a combat sport that involves two competitors directing full force strikes at each other with the hands, elbows, knees, shins, and feet. It is characteristic of dynamic, high-intensity intermittent striking that requires complex skills and tactical excellence for success. Kickboxing training is beneficial to improve strength, muscular power, speed and agility (10), and the effectiveness of training protocols is reproducible in terms of metabolic and technical measurements in young men boxers (11). In addition, kickboxing programs have been found to be beneficial for patients with multiple sclerosis (12), population with different health conditions (13–15), and older adults (16). Although, a few studies in the past have discussed the effects of kickboxing on bone and muscle function (17, 18), none have addressed sarcopenia in community dwelling older people, to date. Therefore, we conducted this randomized controlled trial to investigate the physical effects of kickboxing on parameters of sarcopenia and osteopenia among community-dwelling older people.

## Materials and Methods

### Study Design

This study was designed as a single site randomized controlled trial to determine the effect of a kickboxing training program on sarcopenia and osteoporosis risk profiles. The study was conducted with a 12-week intervention after baseline assessments. The study was approved by the Institutional Review Board of Chang Gung Memorial Hospital, and informed consent was obtained from all of the participants before enrollment.

### Recruitment of Participants

**Figure 1:**
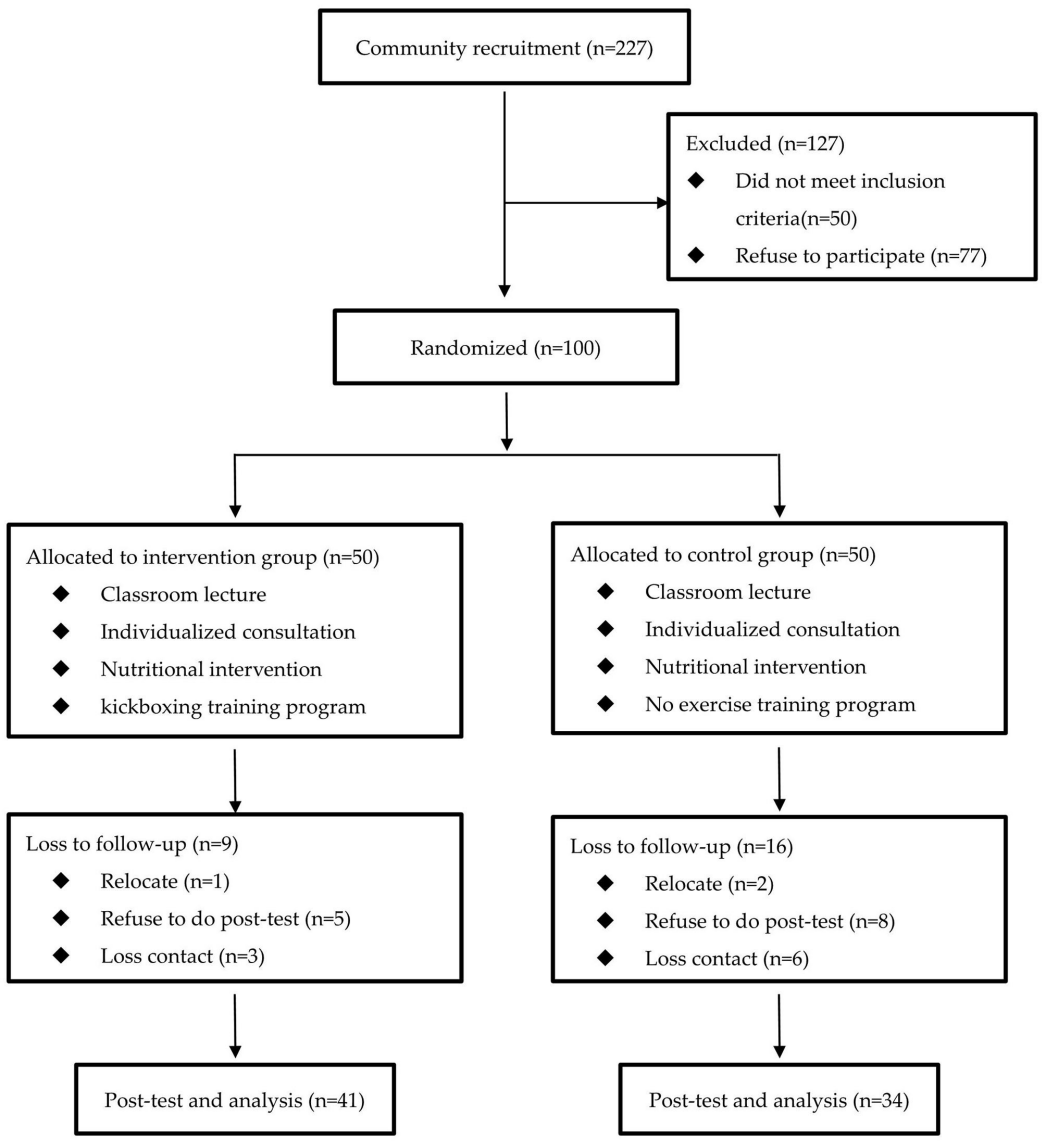
Flow chart of participants who entered the study.

The participants were recruited from the local community around a medical center hospital in the Guishan district in Taoyuan City, Taiwan in 2017. The study protocol was announced with a poster on the bulletin board of the community, and the residents were free to decide whether to participate or not. A total of 227 residents were recruited initially for this health promotion study. The inclusion criteria were, age ranging from 50 to 85 years, the ability to communicate with speech or words, the ability to perform the physical performance tests, absence of chest pain, angina, and arthritis when exercising. The exclusion criteria encompassed severe cardiovascular disease or arthropathy, not being able to walk or maintain balance, difficulty in communicating (e.g., severe cognitive impairment or hearing impairment), medical advice to refrain from exercise, and poorly controlled hypertension (systolic blood pressure >180 mmHg or diastolic blood pressure >120 mmHg) or uncontrolled diabetes mellitus (random blood sugar >300 mg/dl or frequent hypoglycemia symptoms). The study protocol was explained fully and written informed consent was obtained from all the participants prior to initiation. Finally, a total of 100 participants, including 19 men and 81 women, were enrolled in this study, as shown in Figure 1. To determine the sample size of this study, we used G^*^power 3.1 software and set α = 0.05 with a power (1-β) = 0.8 and the effect size difference of 0.75, and it was determined that at least 56 participants would be needed to achieve sufficient statistical power.

### Measurements and Procedures

Baseline assessments were completed before randomization. All the data were assessed again at the end of the intervention (~12 weeks after the baseline assessments). The participants were then randomly assigned to either the intervention group or the control group for a total of 12 weeks. A randomization method that simply involved the participant tossing a coin was used to generate the allocation sequence in our study. Finally, 100 eligible subjects were randomized into an intervention group (*n* = 50) and control group (*n* = 50). Classroom lectures and individualized consultations were conducted biweekly and monthly, respectively, by doctors and nutritionists for both control and experimental groups with regards to issues in sarcopenia, osteoporosis, and related nutrition topics.

We performed anthropometric tests, physical performance tests, and body composition and bone mineral density (BMD) measurements before as well as after the intervention. In addition, all the participants were asked to complete a questionnaire before and after the intervention. Self-reported health status, is assessed on the basis of the question “How is your state of health in general?”, having as optional answers: good, average, and poor. Regular exercise was defined as having the following criteria: “frequency of exercise ≥2 times/week and duration ≥30 min per session”. Height was measured to the nearest 0.1 cm by using a stadiometer, and weight was measured to the nearest 0.1 kg by using electronic scales. BMI (kg/m^2^) was calculated for all the participants. Waist and hip circumference (cm) were measured, and waist-to-height ratio (WHtR) and waist-to-hip ratio (WHR) were calculated. Sarcopenia parameters including handgrip strength (kg) and gait speed (m/s) were also assessed. Handgrip strength was measured by using a grip dynamometer (TKK 5401; Takei Scientific Instruments Co., Ltd., Tokyo, Japan). All the participants received a whole-body scan by using dual-energy X-ray absorptiometry (Hologic Delphi densitometer, Hologic, Waltham, MA, USA). Body composition analysis included total body fat, total lean body mass, total body fat percentage, appendicular lean mass, and visceral fat area. The appendicular skeletal muscle index (ASMI) was also calculated. BMD analysis included the femoral neck bone density (g/cm^2^) and a T-score. At last, a questionnaire including questions about marital status, living status, years of education, self-reported health status, lifestyle, chronic disease status (e.g., hypertension, diabetes and hyperlipidemia), falls in the past 1 year, and chronic pain was completed by every participant.

### Training Program

For the training adaptation purpose, the intervention was divided into phase I (weeks 1 to 5) and phase II (weeks 6 to 12). In the first phase, participants were encouraged to progressively get familiar with the exercise routine by performing seated and standing warm-up exercises for 10 min each. The main kickboxing session was conducted for 40 min followed by body weight training and cool down, each lasting 15 min. In the second phase, starting from standing warm-up for 10 min, the kickboxing session was performed for 50 min, while body weight training and cool down durations remained the same (Table 1). Our non-contact, cardio-based kickboxing practices were implemented in accordance with the general overload and progression training principles, starting from the slower movements and simple directions to better suit the needs of beginner older adults. For safety and quality control, the intervention group was divided into two classes to accommodate reasonable number of participants in each class. The main kickboxing training was conducted using both front stance and combat stance with shadow movements of the pose-jab-cross-uppercut-knee high-and-dodge combination. In the phase II, the main kickboxing content was modified progressively to be more attentive on movement speed, change of direction, balance, and visual challenges. Participants were encouraged to perform in a well-controlled form by focusing on voluntary exertion and movement quality during the training. The program was implemented by a well-trained kickboxing coach. The intervention group participated in weekly 90-min kickboxing sessions, whereas the control group continued with their normal lifestyle during the 12-week study period.

**Table 1:** Training content for main kickboxing exercise.

**Periods**	**Time (mins)**	**Training content**
**Phase I: Week 1–5**		
Main exercise (front stance)	20	Jab-Dodge (1 x 8) Uppercut-Dodge (1 x 8) Jab*4/Uppercut*4 (1 x 8) Forward Jab*4 (1 x 8) Backward Uppercut*4 Forward Jab*4 (1 x 8) -Backward Squat*2 (1 x 8) Forward Uppercut*4 (1 x 8)-Backward Squat*2 (1 x 8)
Main exercise (combat stance)	20	Jab-Cross-Jab-Cross/ Lunge*2 (1 x 8) Jab-Cross-Jab-Cross /Uppercut*2 (2 x 8) Jab-Cross-Jab-Cross /Uppercut*2 (2 x 8) Forward Jab-Cross-Jab-Cross/ Backward (1 x 8) Backward Squat*2 (1 x 8)
**Phase II: Week 6–12**		
Main exercise (front stance)	20	Most were the same as in the first phase and added: Forward double Jab (45°, right-left)-Speed ball (1 x 8) Backward-Squat*2 (1 x 8) Forward double Jab (45°)-Uppercut (right-left) (1 x 8) Backward-Squat*2 (1 x 8) Forward double Jab (45°)-Backward (1 x 8) Squat*2/ Pose (1 x 8) Forward double Jab (45°)-Backward (1 x 8) Uppercut (right-left)*2/ Pose (1 x 8) Forward double Jab (45°)-Backward (1 x 8) Uppercut (right-left)*2/ Squat*2 (1 x 8)
Main exercise (combat stance)	15	Same as in the first phase with adjusted faster movement and shorter time in duration.
Main exercise (combat stance)	15	Forward Jab-Jab-Jab/ Backward (1 x 8) Jab-Cross-Jab-Cross/ Forward Jab *2 (1 x 8) Backward-Pose (1 x 8) Forward Jab-Jab-Jab-Speed ball (1 x 8) Backward Squat*2 (1 x 8) Forward Jab-Jab-Jab-Speed ball (1 x 8) Backward Knee high*2 (1 x 8) Forward Jab-Jab-Jab-Cross (1 x 8) Backward Knee high*2 (1 x 8)

### Definition of Sarcopenia and Osteoporosis

Sarcopenia was defined by EWGSOP (European Working Group on Sarcopenia in Older People) criteria (19), as follows: low muscle mass plus low muscle strength (measured by handgrip strength) or low physical performance (measured by usual walking speed). In this criteria, pre-sarcopenia was defined as low muscle mass, with handgrip strength and gait speed within normal limits. The cut-off values of handgrip strength, gait speed, and muscle mass are derived from the 2014 consensus of AWGS (Asian Working Group for Sarcopenia) (20). According to the consensus, the cutoff values for muscle mass measurements are 7.0 kg/m^2^ for men and 5.4 kg/m^2^ for women when calculated using dual X-ray absorptiometry. The cutoff values for handgrip strength are <26 kg for men and <18 kg for women, and the cutoff value for gait speed is <0.8 m/s in both men and women. By definition of the National Osteoporosis Foundation, osteoporosis was defined as a T score ≤-2.5, whereas osteopenia was defined as a T-score ranging from −1 to −2.5.

### Statistical Analysis

Categorical data were expressed as numbers (percentages), and continuous variables were expressed as means ± SD. Independent Student's *t*-tests and chi-square tests were used to examine the differences between the intervention and control groups at baseline. A paired *t*-test was used to examine the differences before and after the intervention in the intervention and control groups. Analysis of covariance was used to detect intergroup differences. A logistic regression model was used to calculate the odds ratio (OR) of improved and deteriorated parameters in sarcopenia and osteoporosis status following the intervention. All the data analyses were performed using SPSS version 20.0 statistical software (IBM SPSS Statistics; Chicago, IL, USA), and *p* < 0.05 was considered statistically significant.

## Results

Figure 1 presents the flow diagram of participant selection and group allocation followed in the study. We recruited 100 eligible people; 50 were randomized to both the intervention group and the control group. In total, nine people and 16 people were excluded from the intervention and control groups, respectively. Finally, 41 participants in the intervention group and 34 participants in the control group completed the final assessments.

The baseline characteristics of the study population are listed in Table 2. Comparisons of the baseline data between the intervention and control groups, including BMI, waist circumference, WHtR, WHR, physical performance, chronic diseases, geriatric syndromes, bone density, and body composition revealed no significant differences.

**Table 2:** Baseline characteristics of the study population between intervention and control groups.

**Variables**		**Intervention group** **(*n =* 50)**	**Control group** **(*n =* 50)**	**p value**
Basic demographic data				
	Age			0.06
	50–64, n (%)	13 (26.00%)	22 (44.00%)	
	65–85, n (%)	37 (74.00%)	28 (56.00%)	
	Sex (Men)	12 (24.00%)	7 (14.00%)	0.20
	Marital status (single), n (%)	12 (24.00%)	15 (30.00%)	0.50
	Living alone, n (%)	5 (10.00%)	5 (10.00%)	1.00
	Education years			0.44
	≦9, n (%)	42 (84.00%)	39 (78.00%)	
	>9, n (%)	8 (16.00%)	11 (22.00%)	
	Self-reported health status			0.83
	Good, n (%)	15 (30.00%)	16 (32.00%)	
	Fair, n (%)	28 (56.00%)	29 (58.00%)	
	Poor, n (%)	7 (14.00%)	5 (10.00%)	
Lifestyle				
	Current smoker, n (%)	1 (2.00%)	0 (0.00%)	1.00
	Regular exercise, n (%)	45 (90.00%)	45 (90.00%)	1.00
Chronic diseases				
	HTN, n (%)	18 (36.0%)	16 (32.0%)	0.67
	DM, n (%)	12 (24.0%)	6 (12.0%)	0.12
	Hyperlipidemia, n (%)	5 (10.0%)	7 (14.0%)	0.54
Anthropometric measurements				
	Height (cm)	155.78 ± 7.07	154.85 ± 7.41	0.52
	Weight (cm)	58.69 ± 8.35	58.05 ± 7.76	0.69
	Body mass index (g/cm^2^)	24.20 ± 3.20	24.20 ± 2.69	0.99
	Waist circumference (cm)	86.82 ± 8.16	85.35 ± 7.85	0.36
	Hip circumference (cm)	95.70 ± 5.37	95.72 ± 5.54	0.99
	WHtR	0.56 ± 0.05	0.55 ± 0.05	0.58
	WHR	0.91 ± 0.07	0.89 ± 0.07	0.29
Physical performance				
	Hand grip strength (kg)	24.97 ± 6.76	25.92 ± 8.28	0.53
	Gait speed (m/s)	1.34 ± 0.20	1.39 ± 0.27	0.32
Geriatric syndromes				
	Falls in recent 1 year, n (%)	7 (14.0%)	8 (16.0%)	0.78
	Chronic pain, n (%)	11 (22.0%)	12 (24.0%)	0.81
	Sarcopenia status			0.07
	Robust, n (%)	13 (26.0%)	24 (48.0%)	
	Pre-sarcopenia, n (%)	31 (62.0%)	21 (42.0%)	
	Sarcopenia, n (%)	6 (12.0%)	5 (10.0%)	
Dual-energy X-ray absorptiometry				
	Total body fat (kg)	23.36 ± 5.24	23.24 ± 4.35	0.90
	Total lean body mass (kg)	33.46 ± 5.33	32.95 ± 5.36	0.64
	Total body fat (%)	39.62 ± 6.23	40.00 ± 5.16	0.74
	ASMI (kg/m^2^)	5.60 ± 0.76	5.64 ± 0.70	0.75
	Femoral neck BMD (g/cm^2^)	0.61 ± 0.10	0.63 ± 0.10	0.22
	Femoral neck T-score	−2.19 ± 0.88	−1.97 ± 0.94	0.22
	VFA (cm^2^)	136.02 ± 54.83	120.30 ± 38.67	0.10

*Categorical data = n (%); continuous variables = mean ± SD*.

*HTN, Hypertension; DM, diabetes mellitus; WHtR, waist to height ratio; WHR, waist to hip ratio; ASMI, appendicular skeletal muscle index; BMD, bone mineral density; VFA, visceral fat area*.

Table 3 presents the characteristics of the study population before and after the intervention. BMI increased by 1.14% (*p* = 0.002) in the control group, with a significant intergroup change in BMI (*p* = 0.003). The waist circumference and WHtR of the intervention group decreased by 6.54% (*p* < 0.001) and −6.57% (*p* < 0.001), respectively, with significant intergroup differences (*p* < 0.001). Similarly, the WHR of the intervention group decreased by 4.36% (*p* < 0.001) and the intergroup difference was significant (*p* < 0.001). The total body fat decreased by 1.09% (*p* = 0.03) in the intervention group and increased by 1.75% (*p* = 0.01) in the control group with significant intergroup difference (*p* < 0.001). The visceral fat area decreased by 4.6% (*p* = 0.004), with significant intergroup difference (*p* = 0.002).

**Table 3:** Characteristics of the study population who received kickboxing training or control before and after intervention.

**Variables**	**Before the intervention** **(*n =* 75)**	**After the intervention** **(*n =* 75)**	**Percentage difference between groups**	***p* value**	**Intergroup** ***p* value**
SBP (mmHg)					0.47
IG	120.24 ± 16.92	123.61 ± 13.09	2.80%	0.18	
CG	129.38 ± 19.48	125.35 ± 14.66	−3.11%	0.14	
DBP (mmHg)					0.67
IG	71.32 ± 11.60	72.61 ± 9.05	1.81%	0.37	
CG	79.03 ± 10.99	75.97 ± 10.27	−3.87%	0.06	
BMI (kg/m^2^)					0.003
IG	24.13 ± 3.30	24.06 ± 3.26	−0.29%	0.35	
CG	24.06 ± 2.67	24.34 ± 2.61	1.14%	0.002	
Waist circumference (cm)					<0.001
IG	86.28 ± 8.29	80.64 ± 7.29	−6.54%	<0.001	
CG	85.24 ± 8.69	84.78 ± 7.46	−0.53%	0.57	
WHtR					<0.001
IG	0.56 ± 0.06	0.52 ± 0.05	−6.57%	<0.001	
CG	0.55 ± 0.06	0.55 ± 0.05	−0.54%	0.58	
WHR					<0.001
IG	0.90 ± 0.08	0.86 ± 0.06	−4.36%	<0.001	
CG	0.89 ± 0.08	0.90 ± 0.07	0.71%	0.50	
Total body fat (kg)					<0.001
IG	23.43 ± 4.65	23.18 ± 4.38	−1.09%	0.03	
CG	22.84 ± 4.49	23.24 ± 4.47	1.75%	0.01	
Total lean body mass (kg)					0.41
IG	32.58 ± 4.63	32.70 ± 4.62	0.35%	0.46	
CG	33.22 ± 6.06	33.52 ± 6.35	0.90%	0.03	
Total body fat (%)					0.04
IG	40.37 ± 5.41	40.08 ± 5.15	−0.72%	0.09	
CG	39.47 ± 5.54	39.74 ± 5.45	0.69%	0.16	
VFA (cm^2^)					0.002
IG	131.80 ± 50.40	125.74 ± 47.01	−4.60%	0.004	
CG	120.74 ± 40.05	124.64 ± 40.68	3.23%	0.08	
ASMI (kg/m^2^)					0.54
IG	5.52 ± 0.75	5.56 ± 0.77	0.81%	0.15	
CG	5.69 ± 0.77	5.75 ± 0.76	1.17%	0.02	
Hand grip strength (kg)					0.03
IG	24.18 ± 6.24	25.50 ± 6.47	5.46%	0.03	
CG	27.22 ± 9.03	26.65 ± 8.68	−2.11%	0.26	
Gait speed (m/s)					<0.001
IG	1.32 ± 0.18	1.40 ± 0.19	5.71%	0.01	
CG	1.44 ± 0.23	1.32 ± 0.21	−8.48%	<0.001	
Femoral neck BMD (g/cm^2^)					0.003
IG	0.59 ± 0.09	0.59 ± 0.10	0.50%	0.37	
CG	0.63 ± 0.10	0.62 ± 0.10	−1.45%	0.004	

*Categorical data = n (%); continuous variables = mean ± SD*.

*IG, intervention group; CG, control group; SBP, systolic blood pressure; DBP, diastolic blood pressure; BMI, body mass index; HTN, Hypertension; DM, diabetes mellitus; WHtR, waist to height ratio; WHR, waist to hip ratio; ASMI, appendicular skeletal muscle index; BMD, bone mineral density; VFA, visceral fat area*.

Regarding sarcopenia parameters, handgrip strength increased significantly by 5.46% (*p* = 0.03) in the intervention group with significant intergroup difference (*p* = 0.03). The gait speed increased by 5.71% (*p* = 0.01) in the intervention group and decreased 8.48% (*p* < 0.001) in the control group, with significant intergroup difference (*p* < 0.001). The ASMI increased by 0.81% (*p* = 0.15) in the intervention group and by 1.17% (*p* = 0.02) in the control group.

Regarding BMD, the femoral neck BMD and femoral neck T score decreased by 1.45% (*p* = 0.004) and 3.72%, respectively, in the control group, with significant intergroup differences (*p* = 0.003 and *p* = 0.02, respectively).

Table 4 demonstrates that sarcopenia was more likely to improve in the intervention group, with an OR 1.91 (95% CI 0.45–8.10), and less likely to deteriorate, with an OR 0.2 (95% CI 0.02–1.94). In the intervention group, osteoporosis was more likely to improve and less likely to deteriorate, with an OR 0.86 (95% CI 0.12–6.50). However, there were no significant differences between the two groups.

**Table 4:** OR (95% CI) of sarcopenia and osteoporosis status in relation to intervention.

	**Same**			**Improved**			**Deterior**.		
	**n**	**(%)**	**OR**	**n**	**(%)**	**OR**	**n**	**(%)**	**OR**
Sarcopenia									
Intervention group (*n =* 41)	33	(80.49%)	1	7	(17.07%)	1.91	1	(2.44%)	0.20
						(0.45-8.10)			(0.02-1.94)
Control group (*n =* 34)	27	(79.41%)	1	3	(8.82%)	1	4	(11.76%)	1
Osteoporosis									
Intervention group (*n =* 41)	37	(90.24%)	1	2	(4.88%)	-	2	(4.88%)	0.86
									(0.12-6.50)
Control group (*n =* 34)	32	(94.12%)	1	0	(0.00%)	1	2	(5.88%)	1

*Categorical data = n (%)*.

*OR, odds ratio*.

## Discussion

This study was aimed to examine the effect of a 12-week kickboxing intervention on parameters of adiposity, sarcopenia, and osteoporosis in community-dwelling adults aged between 50 and 85 years. Our findings revealed significant improvements in adiposity parameters (e.g., BMI, waist circumference, WHtR, WHR, total body fat, and visceral fat area), sarcopenia parameters (e.g., handgrip strength and gait speed) and osteoporosis parameters (femoral neck BMD and T-scores) in the intervention group.

Our 12-week study of group kickboxing in older community-dwelling adults found improvements in BMI, fat composition, and aerobic capacity (data not shown) in the intervention group. However, the study failed to find improvements in total lean body mass and ASMI. In contrast, previous studies examining the effects of kickboxing on body composition of younger man counterparts have revealed inconsistent results in terms of BMI and body fat (21–24). We are uncertain why lean body mass and the ASMI failed to improve in our study, but several factors may have accounted for this finding including the subject's nutritional status, hormonal balance, injury or disease (25), and the incompliance to exercise training principles, that is, training frequency (26), intensity, and type (25, 27, 28). Another possible reason may be gender dependent bias in body composition, as revealed in a previous meta-analysis where woman gender was found to be associated significantly with decreased skeletal muscle mass index after exercise (28).

Grip strength and walking speed are two major indicators of sarcopenia (20). While walking speed is critically considered as “the sixth vital sign” (29, 30), low muscle strength measured by handgrip has been newly defined as the first characteristic of sarcopenia (6). Interestingly, the loss of muscle strength (i.e., dynapenia) occurs 2–5 times faster than loss of muscle mass (31). Generally, literature supports the role of resistive exercise training in combatting both sarcopenia and dynapenia (31, 32). In the kickboxing intervention group, handgrip strength and gait speed improved without significant increases in total lean body mass or ASMI. Improvement of gait speed may be attributed to the main kickboxing intervention involving various stances, stepping, knee-high movements, and squatting to activate muscles that perform walking (glutei, quadriceps, hamstrings, and gastrocnemius). A previous study by Santos et al., examined the relationship between walking speed and parameters of sarcopenia and dynapenia, and found that the walking speed induced by resistance training was associated with change in body composition, muscle quality, and muscular strength, but not muscle mass, in older women (33). The results of this study coupled with our own results suggest that the change in muscle strength induced by resistance training may precede the change in muscle size, which might be explained by neurological or muscular mechanisms of training (32).

Sarcopenia and osteoporosis shared common risk factors and pathophysiologic mechanisms such as genetic factors, alcohol consumption, smoking, age, sex, and ethnicity. In addition, exercise in older people results in improving muscle mass, physical performance, and bone density. Good nutrition such as adequate calcium and vitamin D intake are beneficial for both bone and muscle mass (34). Biochemical crosstalk between osteoporosis and sarcopenia occurs through myokines and bone-derived factors (35). The effects of exercise and nutrition on osteoporosis and sarcopenia may be due to modulation of both myokines, bone-derived factors, and adipokines from fat (36).

Research has shown that in order to enhance bone mass, it is necessary to place additional forces on the bones beyond the normal weight that is placed on them in daily life (37). In our study, after a 12-week semi-combat cardio-kickboxing training, significant improvements were found in femoral neck BMD and T-score. In a similar interventional study, which investigated the effect of a 12-week yoga or kickboxing program on BMD and serum osteocalcin level in 28 women aged 18–35 years. The results found no significant change on BMD, however, serum osteocalcin level increased, suggesting a positive effect of yoga and kickboxing on bone growth and bone turnover (17). As described formerly, our study experimented with body weight bearing kickboxing exercise. During main training sessions, mechanical stress was adjusted by progressively loading the workout limbs, eliciting varied patterns of strain, not only through voluntary exertion, but also by change of movement acceleration or direction.

Our study demonstrated the effectiveness of a kickboxing training program for improvement in muscle strength, physical performance, and bone density in community-dwelling older adults. Although we did not find a statistically significant increase in muscle mass, the improvement in strength may be of even greater clinical importance. This could be confirmed on the basis of two previous studies, one of which stated that muscle strength is representative of overall health status, and the other concluded that muscle weakness is a more consistent risk for disability and death than muscle mass loss (31, 38). Though our results revealed no significant change in status of sarcopenia and osteoporosis, the change in status tended to favor the intervention group compared with the control group as shown by percentage change and in OR after the intervention. Our findings demonstrate the effectiveness of kickboxing exercise on parameters of sarcopenia and osteoporosis for older adults. Although the results of our study are promising, several limitations should be acknowledged. First, fewer sample size, number of patients lost to follow-up, inability to control subjects' diet and exercise routines, homogenous patient population that limits generalizability, and the subjects also did not report physical activity engagement outside of class, therefore, it may hinder the internal validity of the study. Second, it was not our goal to evaluate the influence of classroom lectures by the doctors and nutritionists on the health behavior and daily dietary intake of the participants. Yet, both the experimental and control groups took the same classes during the study period, which might have minimized their impact on the between-group differences. Third, apart from those who refrained to complete post-test, the average class attendance rate was over 90%. The high motivation of participants may be due to social connection within the group supported by the hospital social service (39). This could limit the applicability of our findings to other populations in different settings. Fourth, Hologic DXA equipment was used both for body composition and bone mineral density (BMD) in the femoral neck but the local precision in repeated measurement was not reported, because BMD usually requires longer time periods for a statistically significant increase. Finally, there was a considerably high percentage of woman subjects (81%) compared with man counterparts in our study. In studies with one gender outnumbering the other, it is a quite common observation that the effect of exercise training on sarcopenic parameters is obscured (28, 40, 41). Therefore, the findings from this study should be cautiously interpreted.

In conclusion, our study demonstrated that a 12-week kickboxing exercise training is an effective option to improve markers of sarcopenia and osteoporosis among community-dwelling older people in Taiwan. We conclude that the semi-combat type of kickboxing exercise is promising to counteract decline of muscle function, and possibly maintain bone health in aging population. In addition, future research is warranted to investigate the gender-specific generalizability of our results in separate studies for different genders with a larger sample size.

## Data Availability Statement

The raw data supporting the conclusions of this article will be made available by the authors, without undue reservation.

## Ethics Statement

The studies involving human participants were reviewed and approved by Chang Gung Medical Foundation Institutional Review Board. The patients/participants provided their written informed consent to participate in this study.

## Author Contributions

L-HC and J-YC: conceptualization and methodology. L-HC, C-CH, and J-YC: validation. J-YC: formal analysis and data curation. Y-AL and J-YC: investigation. Y-AL: writing—original draft preparation. Y-AL and L-HC: writing—review and editing. AW and F-PC: visualization. AW and J-YC: supervision. F-PC and J-YC: project administration and funding acquisition. All the authors have read and agreed to the published version of the manuscript.

## Funding

This study was supported by the Medical Research Center (Chang Gung Memorial Hospital, Keelung, and Chang Gung University), research grants from the Clinical Monitoring Research Program of Chang Gung Memorial Hospital, Keelung (Grant Number CORPG2F0013), and the Health Aging Research Center, Chang Gung University and Taiwan Ministry of Education's Higher Education Deep Plowing Program (Grant Numbers EMRPD1I0411 and EMRPD1I0501) (F-PC). This work was also supported by the Chang Gung Memorial Hospital, grant numbers CORPG3G0021, CORPG3G0022, and CORPG3G0023 (J-YC).

## Conflict of Interest

The authors declare that the research was conducted in the absence of any commercial or financial relationships that could be construed as a potential conflict of interest.

## Publisher's Note

All claims expressed in this article are solely those of the authors and do not necessarily represent those of their affiliated organizations, or those of the publisher, the editors and the reviewers. Any product that may be evaluated in this article, or claim that may be made by its manufacturer, is not guaranteed or endorsed by the publisher.
